# Expression of Concern: COVID-19 infection prevention practices among a sample of food handlers of food and drink establishments in Ethiopia

**DOI:** 10.1371/journal.pone.0329127

**Published:** 2025-07-29

**Authors:** The PLOS One Editors

The *PLOS One* Editors issue an Expression of Concern to alert readers that this article [1] was identified as one of a series of submissions for which we have concerns about authorship and adherence to the journal’s first and second publication criteria. We regret that these issues were not addressed prior to the article’s publication.
