# Supplementary material for: COVID-19 infection prevention practices among a sample of food handlers of food and drink establishments in Ethiopia
Source: PLoS One. 2022 Jan 24;17(1):e0259851. doi: 10.1371/journal.pone.0259851 (PMC8786123; doi:10.1371/journal.pone.0259851)
Supplement: S1 Questionnaires — (DOCX) [file pone.0259851.s001.docx]

## **English version questionnaires**

### Instruction: Please respond to the following questions by encircling or put the answer on the space provided.

**Part I: Socio-demographic variables**

| **S.no** | **Questions** | **Response categories** | Remark |
| --- | --- | --- | --- |
| 1 | Age (in years) | __________________ |  |
| 2 | Sex of the respondent | 1=Male 2 = Female |  |
| 5 | Marital status | 1. Married 2. Single 3. Divorced 4. Widowed |  |
| 4 | Educational status | 1 = Unable to read and write 2 = Informal Education 3 = Primary 4 = Secondary 5 = College or above |  |
| 5 | Job position in the catering establishment | 1. Cook 2. Dish washer 3. Waiter 4. Others/specify___________ |  |
| 6 | Number of service giver | ______________ |  |
| 7 | Years of service | _____________ |  |
| 8 | Monthly income | ____________ |  |

**Part II:** **Availability of supplies**

| **No** | **Questions** | **Responses** | **Remark** |
| --- | --- | --- | --- |
| 1. | Availability of COVID 19 prevention guidelines | 0. No  1. Yes | Interview & Observation |
| 2 | Is there IPC focal person in the establishments | 0. No  1. Yes | Interview |
| 3 | Does your organization have specific budget for PPE in this COVID-19 era? | 0. No  1. Yes | Interview |
| 4. | Availability of personal protective equipment | 1. Glove 2. Mask 3. Boots 4. Gown 5. Burkina 6. Soap 7. Broom 8. Brush 9. Cleaning machine 10. Soft 11. Alcohol   10.sanitizer | Observation |
| 5. | Arrange COVID 19 infection prevention training for food handlers | 0. No  1. Yes | Interview |
| 6. | Posted information related to COVID 19 including emergency phone numbers on visible sites | 0. No  1. Yes | Observation |
| 7. | Provide services in accordance with COVID 19 safety measures | 0. No  1. Yes | Interview |
| 8. | Availability of registration book for documenting events related to COVID 19 | 0. No  1. Yes | Interview & Observation |
| 9. | Availability of visibly posted order for servants and customers to practice social distancing and to avoid touch each other | 0. No  1. Yes | Interview & Observation |
| 10 | Did you link individuals with sign and symptom of COVID 19 to the health institution | 0. No  1. Yes | Interview |
| 11. | Do you wash table cloths, immersing in a mixture of one part Burkina and nine part water for 10 munities and finally rinsing with pure water frequently? | 0. No  1. Yes | Interview |
| 12 | Are services rooms, toilets, meeting halls and corridors well cleaned and ventilated? | 0. No  1. Yes | Observation |
| 13 | Do you clean doors, walls, windows, tables, chairs and mobile phones by sanitizer or a solution containing one part Burkina and nine part water daily? | 0. No  1. Yes | Interview |
| 14 | Do you treat materials and equipments used by customers by a solution containing one part Burkina and nine part water? | 0. No  1. Yes | Interview |
| 15 | Do you rinse brooms, brushes, and utility gloves with Burkina | 0. No  1. Yes | Interview |
| 16 | Is the chair arrangement 2 meters apart? | 0. No  1. Yes | Observation |
|  | **Measures related to waste disposal** |  |  |
| 17 | Accessibility of covered dust bins in each class | 0. No  1. Yes | Observation |
| 18 | Availability of segregating materials for dry and liquid wastes separately | 0. No  1. Yes | Observation |
| 19 | Do you collect and dispose wastes properly | 0. No  1. Yes | Interview & Observation |

**Part III. Knowledge about IPC implementation for COVID-19**

| Q No | Interview question | Response | Comment |
| --- | --- | --- | --- |
| 1 | All microorganisms including corona viruses are removed by washing with water and antibiotic agents | 0. No  1.Yes |  |
| 2 | All people are at risk of COVID-19? | 0. No  1.Yes |  |
| 3 | Washing hand frequently with soap, sanitizer and water would physically remove, inhibit or kill corona virus from hands | 0. No  1.Yes |  |
| 4 | There is a need to wash hands before and after touching things | 0. No  1.Yes |  |
| 5 | Being 2 meter far apart from individual prevents the transmission of COVID-19 | 0. No  1.Yes |  |
| 6 | The incubation period of corona virus is 14 days | 0. No  1.Yes |  |
| 7 | Wearing PPEs ( like mask, goggle, glove...) decreases the risk of transmission of COVID-19. | 0. No  1.Yes |  |
| 8 | Cloth mask can be reused after washing with soap and water, decontaminating and dry but not surgical mask | 0. No  1.Yes |  |
| 9 | Wearing gloves does not replace the need for hand washing or use of antiseptic hand rubs | 0. No  1.Yes |  |
| 10 | It is not recommended to touch nose, mouth, eye even if you done glove when you give service | 0. No  1.Yes |  |
| 11 | Surgical gloves can be reused | 0. No  1.Yes |  |

**Part IV: Attitude toward IPC implementation for COVID-19**

| Q No | Questions | Responses | Comment |
| --- | --- | --- | --- |
| 1 | Washing hands with soap or an alcohol based antiseptic decreases the risk of transmission of COVID-19 | 1. Strongly Disagree  2. Disagree  3. Neutral  4. Agree  5. Strongly agree |  |
| 2 | Gloves and mask provide complete protection against COVID-19 | 1. Strongly Disagree  2. Disagree  3. Neutral  4.Aagree  5. Strongly agree |  |
| 3 | Hand washing is unnecessary when gloves are worn | 1. Strongly Disagree  2. Disagree  3. Neutral  4. Agree  5. Strongly agree |  |
| 4 | Frequent hand washing damages skin and causes cracking, dryness, irritation and dermatitis. | 1. Strongly Disagree  2. Disagree  3. Neutral  4. Agree  5. Strongly agree |  |
| 5 | You have a very low risk of acquiring COVID-19 from others | 1. Strongly Disagree  2. Disagree  3. Neutral  4. agree  5. Strongly agree |  |
| 6 | COVID-19 is like common cold which has no serious effect | 1. Strongly Disagree  2. Disagree  3. Neutral  4. Agree  5. Strongly agree |  |
| 7 | Gloving is a useful strategy for reducing risk of transmission novel Corona virus | 1. Strongly Disagree  2. Disagree  3. Neutral  4. Agree  5. Strongly agree |  |
| 8 | Social distancing is a basic technique to reduce the transmission novel Corona virus | 1. Strongly Disagree  2. Disagree  3. Neutral  4. agree  5. Strongly agree |  |
| 9 | Being locked down prevents the transmission novel Corona virus | 1. Strongly Disagree  2. Disagree  3. Neutral  4. Agree  5. Strongly agree |  |

**Part V. Food handlers’ IPC strategy practice for COVID-19**

| Q No | Interview question | Response | Comment |
| --- | --- | --- | --- |
| 1 | Do you wash your hand regularly | 0. No  1.Yes | If no skip to q5 |
| 2 | When do you wash your hands? (it is possible to circle more than one item) | 1. Before Contact of things 2. After Contact of things 3. Before preparing food 4. After preparing food 5. If I look or feel dirty 6. Before going to outside of the home 7. Before entering to the home from outside 8. Before going to the toilet 9. After going to the toilet 10. Before donning gloves 11. After removing gloves   H. After sneezing |  |
| 3 | Would you show me how to wash your hand practically please | 110. Not show perfectly  221. Show perfectly  2 | Observation |
| 4 | What do you use to wash your hand? (it is possible to circle more than one answer ) | 1. With water only  2. With plain Soap and water  3. With anti-bacterial Soap and water  4. With Alcohol/Sanitizer  5. Other specify_______ |  |
| 5 | If your answer is no for question no 1 why you did not wash your hands? | 1. Have no information how to wash hand 2. Unavailability of hand washing materials 3. Negligence 4. Other specify_______ |  |
| 6 | Do you use antiseptic hand rub? | 0. No  1.Yes |  |
| 7 | Do you wear personal protective equipments to prevent COVID-19? | 0. No  1.Yes | If question No 407 is no skip to question 409 |
| 8 | If your answer is yes for question no 6, which type(it is possible to circle more than one item) | 1. Gloves  2. Gown  3. Caps  4. Goggle  5. Mask  6. Other specify_______ |  |
| 9 | If no, why you are not use personal protective equipment? | 1. Lack of materials 2. Lack of awareness 3. Difficult to work with 4. Not always necessary 5. Carelessness 6. Other (specify)----------- |  |
| 10 | Do you sneeze by covering with your elbow? | 0. No  1.Yes |  |
| 11 | Do you touch your nose, mouth and eye with your hand when you host customers? | 0. No  1.Yes |  |
| 12 | Do you keep your social distance that is 1 meter apart? | 0. No  1.Yes |  |
| 13 | Do you take shower and change cloth before contacting with your family | 0. No  1.Yes |  |
| 14 | Do you use infection prevention and control guidelines for COVID-19 in your working area? | 0. No  1.Yes |  |
| 15 | Have you ever taken training about IPC of Covid-19 era? | 0. No  1.Yes |  |

**Thank you for your valuable information and participation!!!**
