# Supplementary material for: COVID-19 infection prevention practices among a sample of food handlers of food and drink establishments in Ethiopia
Source: PLoS One. 2022 Jan 24;17(1):e0259851. doi: 10.1371/journal.pone.0259851 (PMC8786123; doi:10.1371/journal.pone.0259851)
Supplement: S2 Questionnaires — (DOCX) [file pone.0259851.s002.docx]

## **የአማርኛ ትርጉም መጠይ**

## **መመሪያ:** **እርስዎ ያለዎትን አማራጭ በመያዝ እባክዎን ለሚቀጥሉት ጥያቄዎች መልስ ይስጡ**

**ክፍል 1**፡- ማህበራዊና ስነ-ህዝባዊ ገጽታወችን የተመለከቱ ጥያቄዎች

| ኮድ | መጠይቅ | ምርጫ | ምርመራ |
| --- | --- | --- | --- |
| 1 | እድሜ (በሙለ አመት) | __________________ |  |
| 2 | ፆታ | 1. ወንድ 2. ሴት |  |
| 3 | የጋብቻ ሁኔታ | 1. ያገባ/ች 2. ያላገባ/ች 3. የፈታ/ች 4. የሞተበት/ባት |  |
| 4 | የትምህርት ደረጃ | 1. ማንበብና መፃፍ የማይችል 2. ኢ-መደበኛ ትምህርት የተማረ 3. የመጀመሪያ ደረጃ ትምህርት የተማረ 4. የሁለተኛ ደረጃ ትምህርት የተማረ 5. ኮሌጅ ወይም ከዚያ በላይ የተማረ |  |
| 5 | የስራ ድርሻ | 1. ማብሰል 2. ዕቃዎችን ማፅዳት 3. አስተናጋጅ 4. ሌላ |  |
| 6 | የሰራተኛ ብዛት | ______________ |  |
| 7 | የአገልግሎት ዓመታት | _____________ |  |
| 8 | ወርሃዊ ገቢ | ____________ |  |

**ክፍል 2**፡- **አቅርቦትን የተመለከቱ መጠይቆች**

| ኮድ | መጠይቅ | ምርጫ | ምርመራ |
| --- | --- | --- | --- |
| 1. | የኮቪድ-19 መከላከያ መመሪያ ከምግብ እና መጠጥ ቤታችሁ ይገኛል? | 0. የለም 1. አለ | ቃለ ምልልስ እና ምልከታ |
| 2 | ከምግብ እና መጠጥ ቤታችሁ ውስጥ ስለ ኢንፌክሽን መከላከል እና ቁጥጥር የሚከታተል ሀላፊ አለ? | 0. የለም 1. አለ | ቃለ ምልልስ |
| 3 | ድርጅትዎ በዚህ ኮቪድ -19 ወረርሽኝ ዘመን ኢንፌክሽን ለመከላከል የተወሰነ በጀት አለዉ? | 0. የለም 1. አለ | ቃለ ምልልስ |
| 4. | በድርጅቱ ዉስጥ የመከላከያ መሣሪያዎች ተደራሽነት | 1. ጓንት 2. ጭምብል 3. ቦት ጫማዎች 4. ጋዋን 5. በረኪና 6. ሳሙና 7. መጥረጊያ 8. ብሩሽ 9. የማፅጃ ማሽን 10. ሶፍት 11. አልኮል   10.ሳኒታይዘር | ምልከታ |
| 5. | ድርጅትዎ ስለ ኮቪድ -19 ወረርሽኝ መከላከል ስልጠና እንዲወስዱ ያመቻቻል? | 0. የለም 1. አዎ | ቃለ ምልልስ |
| 6. | ኮቪድ -19 ወረርሽኝን ለማሳወቅ ከሚታይ ቦታ ላይ የተለጠፉ መረጃዎች አሉ ለምሳሌ ስልክ ቁጥር? | 0. የለም 1. አዎ | ምልከታ |
| 7. | የኮቪድ -19 የደህንነት እርምጃዎችን መሠረት ያደረገ አገልግሎት ይሰጣል? | 0. የለም 1. አዎ | ቃለ ምልልስ |
| 8. | ከኮቪድ -19 ጋር የተያያዙ መረጃዎችን ለመመዝገብ የሚያገለግል የምዝገባ አጀንዳ አለ? | 0. የለም 1. አዎ | ቃለ ምልልስ እና ምልከታ |
| 9. | ሰራተኞች እና ደንበኞች ርቀታቸዉን እንዲጠብቁ እና ንክኪ እንዲያስዎግዱ የሚያስረዳ ፖስተር በሚታይ ሁኔታ ተለጥፉል? | 0. የለም 1. አዎ | ቃለ ምልልስ እና ምልከታ |
| 10 | የኮቪድ -19 ምልክት ያላቸውን ግለሰቦች ከጤና ተቋም ጋር ያገናኙቸዋል? | 0. የለም 1. አዎ | ቃለ ምልልስ |
| 11. | የጠረጴዛ ልብሶች በየጊዜዉ ታጥበዉ ለ 10 ደቂቃ በበረኪና ይዘፈዘፋሉ እንድሁም በንጹህ ዉሃ ይለቃለቃሉ? | 0. የለም 1. አዎ | ቃለ ምልልስ |
| 12 | የመስተንግዶ ክፍሎች, መጸዳጃ ቤቶች, የስብሰባ አዳራሾች እና ኮሪደሮች ንጹህ እና በቂ አየር የሚደርሳቸዉ ናቸዉ? | 0. የለም 1. አዎ | ምልከታ |
| 13 | በሮችን, ግድግዳዎችን, መስኮቶን እና የሞባይል ስልኮችን በየዕለቱ በበረኪና ያፀዳሉ? | 0. የለም 1. አዎ | ቃለ ምልልስ |
| 14 | ደንበኞች የሚጠቀሙባቸውን ቁሳቁሶች በበረኪና ያፀዳሉ? | 0. የለም 1. አዎ | ቃለ ምልልስ |
| 15 | መጥረጊያወችን፡ ብሩሾችን: እና የመገልገያ ጓንትዎን በበረኪና ያጥባሉ? | 0. የለም 1. አዎ | ቃለ ምልልስ |
| 16 | የወንበሮች የርስ በርስ ርቀት 2 ሜትር ይሆናል? | 0. የለም 1. አዎ | ምልከታ |
|  | **ከቆሻሻ አወጋገድ ጋር የተዛመዱ መረጃዎች** |  |  |
| 17 | በእያንዳንዱ ክፍል ውስጥ ክዳን ያለዉ የቆሻሻ ማስዎገጃ እቃ አለ? | 0. የለም 1. አዎ | ምልከታ |
| 18 | የደረቅ እና ፈሳሽ ቆሻሻ ማስዎገጃ እቃወች የተለያዩ ናቸዉ? | 0. የለም 1. አዎ | ምልከታ |
| 19 | ቆሻሻን በአግባቡ ያስወግዳሉ? | 0. የለም 1. አዎ | ቃለ ምልልስ እና ምልከታ |

**ክፍል 3፡- ኮቪድ -19 ወረርሽኝ መከላከልን በተመለከተ የምግብ ቤት ሰራተኞች ያላቸው እውቀት**

| ኮድ | መጠይቅ | ምርጫ | ምርመራ |
| --- | --- | --- | --- |
| 1 | ሁለም ረቂቅ ተዋህስያን ኮሮና ቫይረስን ጨምሮ እጅን በሳሙና እና በዉሃ በመታጠብ ይወገዳሉ፡፡ | 0. ስህተት 1.ትክክል |  |
| 2 | ሁሉም ሰዎች ለኮቪድ -19 ተጋላጭ ናቸዉ:: | 0. ስህተት 1.ትክክል |  |
| 3 | አጅን በሳሙናና ዉሃ፡ በሳኒታይዘር መታጠብ ኮሮና ቫይስን ከእጅ ይገድባል፡ ያስወግዳል ወይም ይገድላል:: | 0. ስህተት 1.ትክክል |  |
| 4 | ነገሮችን ከመነካካት በፊት እና በኋላ እጅን መታጠብ አስፈላጊ ነው | 0. ስህተት 1.ትክክል |  |
| 5 | ከግለሰብ 2 ሜትር መራቅ የኮቪድ -19 ተጋላጭነትን ይከላከላል፡: | 0. ስህተት 1.ትክክል |  |
| 6 | ኮሮና ቫይረስ ምልክት የሚያሳየዉ ከ14 ቀናት በሁላ ነው፡: | 0. ስህተት 1.ትክክል |  |
| 7 | የግል መከላከያ መሳሪያዎችን መልበስ (እንደ ጭምብል:መነፅር:ጓንት ...)) የኮቪድ-19 ን የመተላለፍ መጠን ይቀንሳል:: | 0. ስህተት 1.ትክክል |  |
| 8 | የጨርቅ ጪምብል በሳሙና ከታጠበ እና ከደረቀ በሁላ እንደገና መጠቀም ይቻላል: ሰርጅካል ጪምብልን ግን አንዴ ከተለበሰ እንደገና መልበስ አይቻልም፡፡ | 0. ስህተት 1.ትክክል |  |
| 9 | ጓንት መልበስ እጅ መታጠብን ወይም ሳኒታይዘር መጠቀምን አይተካም፡: | 0. ስህተት 1.ትክክል |  |
| 10 | አገልግሎት በሚሰጡበት ጊዜ ጓንት ቢያደርጉም እንኳ አፍንጫን:አፍን እና ዓይን መንካት አይመከርም፡: | 0. ስህተት 1.ትክክል |  |
| 11 | የቀዶ ጥገና ጓንቶች እንደገና ጥቅም ላይ ሊውሉ ይችላሉ፡: | 0. ስህተት 1.ትክክል |  |

**ክፍል 4፡- ኮቪድ -19 ወረርሽኝ መከላከልን በተመለከተ የምግብ ቤት ሰራተኞች ያላቸው አመለካከት**

| ኮድ | መጠይቅ | ምርጫ | ምርመራ |
| --- | --- | --- | --- |
| 1 | እጅን በሳሙና ወይም በእጅ ማከሚያ አልኮል መታጠብ የኮሮና ቫይስን ስርጭት ለመከላከል ይጠቅማል፡፡ | 1. በጣም አልስማማም  2.አልስማማም  3.እርግጠኛ አይደለሁም  4. እስማማለሁ  5. በጣም እስማማለሁ |  |
| 2 | ጓንቶች እና ጭምብሎችን መጠቀም ኮቪድ -19ን ሙሉ በሙሉ ይከላከላል፡፡ | 1. በጣም አልስማማም  2.አልስማማም  3.እርግጠኛ አይደለሁም  4. እስማማለሁ  5. በጣም እስማማለሁ |  |
| 3 | ጓንት በሚለብሱበት ጊዜ እጅ መታጠብ አስፈላጊ አይደለም፡፡ | 1. በጣም አልስማማም  2.አልስማማም  3.እርግጠኛ አይደለሁም  4. እስማማለሁ  5. በጣም እስማማለሁ |  |
| 4 | በተደጋጋሚ እጅን መታጠብ ቆዳን ያደርቃል እንዲሁም የቆዳ በሽታን ያመጣል፡፡ | 1. በጣም አልስማማም  2.አልስማማም  3.እርግጠኛ አይደለሁም  4. እስማማለሁ  5. በጣም እስማማለሁ |  |
| 5 | እርስዎ ኮቪድ -19 ን ከሌላው ሰዉ የመያዝ እድልዎ ዝቅተኛ ነዉ፡፡ | 1. በጣም አልስማማም  2.አልስማማም  3.እርግጠኛ አይደለሁም  4. እስማማለሁ  5. በጣም እስማማለሁ |  |
| 6 | ልክ እንደ ጉንፋን ኮቪድ -19ም ከባድ ጉዳት የለውም፡፡ | 1. በጣም አልስማማም  2.አልስማማም  3.እርግጠኛ አይደለሁም  4. እስማማለሁ  5. በጣም እስማማለሁ |  |
| 7 | ጓንት መልበስ የኮሮና ቫይረስን የመተላለፍ አቅም ለመቀነስ ከፍተኛ ጥቅም አለዉ፡፡ | 1. በጣም አልስማማም  2.አልስማማም  3.እርግጠኛ አይደለሁም  4. እስማማለሁ  5. በጣም እስማማለሁ |  |
| 8 | ርቀትን መጠበቅ የኮሮና ቫይረስን የመተላለፍ አቅም ለመቀነስ ቁልፍ ዘዴ ነዉ፡፡ | 1. በጣም አልስማማም  2.አልስማማም  3.እርግጠኛ አይደለሁም  4. እስማማለሁ  5. በጣም እስማማለሁ |  |
| 9 | ከቤት መቀመጥ የኮሮና ቫይረስን ስርጭት ይቀንሳል፡፡ | 1. በጣም አልስማማም  2.አልስማማም  3.እርግጠኛ አይደለሁም  4. እስማማለሁ  5. በጣም እስማማለሁ |  |

**ክፍል 5፡- የምግብ ቤት ሰራተኞች የኮቪድ -19 ወረርሽኝን የመከላከል ትግበራ መጠይቆች**

| ኮድ | መጠይቅ | ምርጫ | ምርመራ |
| --- | --- | --- | --- |
| 1 | እጅዎን በመደበኛነት ይታጠባሉ? | 1. የለም 2. አዎ | ለጥያቄ ቁጥር 1 መልስዎ የለም ከሆነ ወደ ጥያቄ ቁጥር 5 ይለፉ |
| 2 | መቼ መቼ ነው እጅዎትን የሚታጠቡት?(ከአንድ በላይ መልስ መመለስ ይቻላል) | 1. ነገሮችን ከመነካካቴ በፊት 2. ነገሮችን ከነካሁ በኋላ 3. ምግብ ከማዘጋጀቴ በፊት 4. ምግብ ካዘጋጅሁ በኋላ 5. እጆቸ ቆሽሸዋል ብየ ካሰብሁ 6. ወደ ዉጭ ከመዉጣታችን በፊት 7. ወደ ቤት ከመግባታችን በፊት 8. መጸዳጃ ቤት ከመሄዳችን በፊት 9. ከመጸዲጃ ቤት መልስ 10. ጓንት ከመልበሳችን በፊት 11. ጓንት ካወለቅን በኋላ 12. ካስነጠስን በኋላ |  |
| 3 | እጅዎን እንዴት እንደሚታጠቡ በተግባር አሳዩኝ? | 11 0. በትክክል አላሳዩም  22 1. በትክክል ኣሳይተዋል  2 | ምልከታ |
| 4 | እጅዎን ለመታጠብ ምን ይጠቀማሉ? (ከአንድ በላይ መልስ መምርጥ ይቻላል) | 1. ዉሃ ብቻ  2. ዉሃ እና ሳሙና  3. ዉሃ እና ፀረ-ተህዋሲያን ያለዉ ሳሙና  4. አልኮል/ሳኒታይዘር  5. ሌላ |  |
| 5 | ለጥያቄ ቁጥር 1 መልስዎ የለም ከሆነ ለምንድን ነዉ የማይታጠቡት? | 1. እንዴት እንደምታጠብ መረጃ የለኝም 2. የእጅ መታጠቢያ ቁሳቁችን አላገኝም 3. ቸልተኝነት 4. ሌላ |  |
| 6 | እጅዎን በፀረ-ተባይ ኬሚካል ይታጠባሉ? | 1. የለም 2. አዎ |  |
| 7 | ኮቪድ -19 ን ለመከላከል የግል መከላከያ ቁሳቁሶችን ትለብሳሉ? | 1. የለም 2. አዎ | ለጥያቄ ቁጥር 7 መልስዎ የለም ከሆነ ወደ ጥያቄ ቁጥር 9 ይለፉ |
| 8 | መልስዎ አዎ ከሆነ , የትኛው ዓይነት (ከአንድ በላይ እቃዎችን መምረጥ ይቻላል) | 1. ጓንት  2.ጋዋን  3. ኮፍያ  4. መነፅር  5.ጭምብል  6. ሌላ |  |
| 9 | ለጥያቄ ቁጥር 7 መልስዎ የለም ከሆነ, ለምንድን ነዉ የማይለብሱት? | 1. የቁሳቁስ እጥረት 2. የግንዛቤ ማጣት 3. ለብሶ ለመስራት አስቸጋሪ ስለሆነ 4. ሁልጊዜ አስፈላጊ አይደለም 5. ግድየለሽነት 6. ሌላ |  |
| 10 | አፍንጫዎንና አፍዎን በክርንዎ በመሸፈን ነዉ የሚስነጥሱ? | 1. አድለም 2. አዎ |  |
| 11 | ደንበኞችን ሲያስተናግዱ አፍንጫዎን, አፍዎን እና ዓይንዎን በእጅዎ ይነካሉ? | 1. የለም 2. አዎ |  |
| 12 | ማህበራዊ ርቀትዎን 1 ሜትር እንዲሆን ይጠብቃሉ? | 1. የለም 2. አዎ |  |
| 13 | ከቤተሰብዎ ጋር ከመገናኘትዎ በፊት ገላዎን ታጥበዉ ጨርቆትን ይለውጣሉ? | 1. የለም 2. አዎ |  |
| 14 | በስራ ቦታዎ ውስጥ የኮቪድ -19 መከላከል እና ቁጥጥር መመሪያዎችን ይጠቀማሉ? | 1. የለም 2. አዎ |  |
| 15 | በኮቪድ -19 ወረርሽኝ ዘመን ስለ ኮሮና ቫይረስ መከላከል እና መቆጣጠር ስልጠና ወስደዉ ያዉቃሉ? | 1. የለም 2. አዎ |  |

**ስለተሳትፎዎ እናመሰግናለን !!!**
