# Supplementary material for: COVID-19 infection prevention practices among a sample of food handlers of food and drink establishments in Ethiopia
Source: PLoS One. 2022 Jan 24;17(1):e0259851. doi: 10.1371/journal.pone.0259851 (PMC8786123; doi:10.1371/journal.pone.0259851)
Supplement: Attachment — Submitted filename: Response to reviwers.docx [file pone.0259851.s004.docx]

**Response to reviewers to for the rejected paper PONE-D-20-33577**

**COVID-19 Infection Prevention and Control Practices among Food Handlers in Food and Drinking Establishments of Dessie City and Kombolcha Towns in Ethiopia**

Reviewer #1: The manuscript should be considered for following changes

**Response: Dear reviewer. Thank you for your valuable comments and we updated the manuscript using reviewer 1 and II your comments.**

**Here is the detail answers for your concerns:**
1- Why the sample size is different among two selected study area i.e, (349 & 204) Dessie and Kombolcha towns?

**Response: Thank you for your concern. The points the reviewer mentioned 349 and 204 are not sample size, rather it is the total number of food and drinking establishment in Dessie and Kombolcha town, respectively. SO the reviewer miss understood our paper. However, the actual sample size for Dessie and Kmobolcha was 266 and 156. This sample size varied due to the fact that the total number of food and drinking establishments varied among the two towns. Here is how we determined sample size and how we proportionally allocated sample size.**

**The sample size was determined using single population proportion formula with the assumptions of expected good IPC practice to prevent COVID-19 among food handlers 50% due to the absence of previous studies in a similar setting, Z_α/2_ value 1.96 at 95% confidence interval (CI) and 5% margin of error. After considering a 10% non-response rate from the calculated sample size of 384, the final sample size was 422. The total sample size was proportionally allocated for Dessie and Kombolcha food establishments based on the total number of food establishments for each town. There were 349 and 204 food establishments (hotel, restaurant, bar and restaurant, cafeteria, and butcher houses) in Dessie and Kombolcha respectively. Therefore, the numbers of food establishments from the two towns by proportional allocation were 266 food establishments from Dessie and 156 from Kombolcha.**

2- Did you already get information regarding education status of food handlers and choose on that behalf sample size?

**Response: The education status was obtained after the study participant selected. However, prior to the selection of the study participant we did not know about the education status of the study participant. The sample size has no relation with education status since we determined the sample size using the outcome variables of infection prevention and control practices (Please see the sample size determination in the updated manuscript). The sample size was determined using single population proportion formula with the assumptions of expected good infection prevention and control practice to prevent COVID-19 among food handlers 50% due to the absence of previous studies in a similar setting, Z_α/2_ value 1.96 at 95% confidence interval (CI) and 5% margin of error. After considering a 10% non-response rate from the calculated sample size of 384, the final sample size was 422.**

3- Face to face interviewer-administered questionnaire? Is it appropriate to get info without prior knowledge or information sharing?

**Response: Yes, data collection using Face to face interviewer-administered questionnaire means that the data collector interview directly the study participant. Since from one food and drinking establishment only one food handler is part of the study, there will be no information sharing within the same food establishment due to others are not part of the study. To minimize ambiguity for readers, we revised the data collection as data were collected using a structured pre-tested questionnaire and an on-the-spot observational checklist. The questionnaire and observational checklist were adapted from various reviewed literature, such as WHO and EMOH guidelines about COVID-19 IPC [7, 13, 17, 22]. The prepared English questionnaire was translated to Amharic (a local language) and then retranslated back to English by bilingual experts to ensure its consistency (See in updated version).**

4- The manuscript has English language errors that must be removed e.g., see line 1st of data collection and quality control heading.

**Response: We got language editing service from the first English language speaker. We acknowledged the editor in the acknowledgement section as follows. The last but not the least we also thank Lisa Penttila for language editing of the manuscript.**

5- Overall, manuscript is not well planned to justify the outcomes. There are flaws in questionnaire design and overall implementation that must be justified.

**Response: Sorry for the error. We re-write the methods section and please see the data collection procedures and sampling methods we sued. Please also see how we design the questionnaire. We hope that the revised version is acceptable.**

Reviewer #2: the paper does not contain any scientific information about the COVID-19. Method of data collection and selection of the participant was not in scientific manner. All data collection looks ambiguous, same data presented many times in paper.

**Response: The selection of the study participant and method of data collection and revised and we hope that now it becomes very clear. The sampling units were food and drinking establishments that had at least one food handlers whose age 18 years and above. Sampling frame and source population was prepared by taking a list of food and drinking establishments that have at least food handlers (18 years and above) to be included in the sampling frame. Then, those eligible food and drinking establishments were coded to easily differentiate them during house-to-house visits at the time of data collection. Then, based on the source population (food handlers) for each *kebele,* sample size was proportionally allocated for all ten *kebeles* based on their number of size of the food and drinking establishments. Using the sampling frame, a systematic random sampling with a fixed interval of every house was used to select sampling unit within each *kebele.* To select the house-paired with one eligible study participant, the data collector started with a bench mark of the known location and then walked straight forward to identify each house.**

**Furthermore, when more than one eligible study participant was present in the selected food and drinking establishments, a lottery method was used to select one study participant to estimate the proportion of good or poor practice of infection prevention and control in the study area relative to the total sample size. When one eligible study participant was not available from the selected food and drinking establishments, another visit was made the same day. If they were again not available, another visit was performed the next day in order to minimize the non-response rate. If not available after the three visits, it was taken as non-respondent (Please see the revised version of the sampling procedure).**

**We hope that the revised version of sampling methods mentioned above for the data collection is enough as scientific quality is concerned. We revised the data presentation in a good manner by avoiding repetitions (Please see Tables 1, 2 and 3) and Figures 1, 2 and 3)**

**Thank you for your time in reviewing our paper and we hope that the revised version is acceptable for publication in PLoS ONE.**

**Sincerely yours,**

**Dr Metadel Adane (PhD in Water and Public Health)**
