# Supplementary material for: COVID-19 infection prevention practices among a sample of food handlers of food and drink establishments in Ethiopia
Source: PLoS One. 2022 Jan 24;17(1):e0259851. doi: 10.1371/journal.pone.0259851 (PMC8786123; doi:10.1371/journal.pone.0259851)
Supplement: Attachment — Submitted filename: Manuscript_Review.docx [file pone.0259851.s005.docx]

**COVID-19 Infection Prevention and Control Practices among Food Handlers of Food and Drinking Establishments of Dessie City and Kombolcha Towns in Northeastern Ethiopia**

Atsedemariam Andualem^1^, Belachew Tegegne^1^, Sewunet Ademe^1^, Tarikuwa Natnael^2^, Gete Berihun^2^, Masresha Abebe^2^, Yeshiwork Alemnew^3^, Metadel Adane^2*^

^1^Department of Nursing, School of Nursing and Midwifery, College of Medicine and Health Sciences, Wollo University, Dessie, Ethiopia

^2^Departments of Environmental Health, College of Medicine and Health Sciences, Wollo University, Dessie, Ethiopia

^3^Department of Biology, College of Natural Sciences, Wollo University, Dessie, Ethiopia

*metadel.adane2@gmail.com

# **Abstract**

## **Background**

Cases of coronavirus infectious disease-19 (COVID-19) are increasing at an alarming rate throughout the world, including Ethiopia. Food handlers are highly exposed to the virus due to their many daily contacts with customers visiting their food and drinking establishments. Thus, ensuring infection prevention and control practices for this high-risk group is urgently required to prevent COVID-19 transmission. Since there is a paucity of information about infection prevention and control practices among food handlers in the Ethiopian urban of Dessie City and Kombolcha Towns. This study was designed to address this gap.

## **Method**

A facility-based cross-sectional study design was conducted among 422 food handlers in Dessie City and Kombolcha Town food and drinking establishments in July and August 2020. The study participants were selected using a simple random sampling technique. Data were collected by trained data collectors using a pre-tested structured questionnaire and an on-the-spot observational checklist. The collected data were entered into EpiData version 4.6 and exported to statistical package for social sciences (SPSS) version 25.0 for data cleaning and analysis. Data were analyzed using bivariate and multivariable logistic regression model at 95% confidence interval (CI). Variables that had *a p*-value < 0.25 from the bivariate analysis were retained in multivariable logistic regression analysis for controlling confounders. From the multivariable analysis, variables that had a *p*-value < 0.05 were taken as factors significantly associated with good infection prevention and control practices to prevent COVID-19.

## **Main findings**

The overall rate of good practice of infection prevention and control among food handlers was 43.9% (95% CI: 38.9%-48.6%), whereas the poor practice of infection prevention and control was 56.1% (95% CI: 51.4%-60.8%). Of the total 401 food handlers, 78.1% had good knowledge and 58.4% had a favourable attitude about the infection prevention and control of COVID-19. Factors significantly associated with good infection prevention and control practices to prevent COVID-19 were: educational status of the college or above (AOR=1.97; 95% CI:1.32–3.75), food handling work experience greater than five years (AOR=2.55; 95% CI:1.43-5.77), availability of written guidelines within food and drinking establishments (AOR=2.68; 95% CI:1.52-4.75), taking training about infection prevention and control (AOR=3.26; 95% CI:1.61-6.61).

## **Conclusion**

Just more than one-third of food handlers had good infection prevention and control practices. Our findings also showed that three-fourths of food handlers had good knowledge and more than half had a favourable attitude about infection prevention and control. Thus, integrated work is urgently needed to prevent COVID-19 transmission by improving food handlers’ good practice of infection prevention and control, knowledge, and attitude about infection prevention and control through providing health education, and training about infection prevention and control by making written infection prevention and control guidelines available in food and drinking establishments.

**Keywords:** Infection Prevention and Control Practices; Food Handlers; COVID-19 Transmission; Northeastern Ethiopia

# **Introduction**

Coronavirus infectious disease-19 (COVID-19) is a global public health threat caused by severe acute respiratory syndrome coronavirus-2 (SARS-CoV-2) that was first reported in Wuhan, China in December 2019 and has now spread throughout countries and territories outside of China[1, 2]. COVID-19 is the most overwhelming problem of the 21^st^ century across the world that results in morbidity and mortality ranging from mild respiratory illness to severe acute respiratory distress syndrome, septic shock, and other metabolic and homeostasis disorders and death [3]. Even though COVID-19 affects the whole population, the most fatal acute respiratory distress syndrome (ARDS) has occurred in older adults and people who have existing chronic medical conditions like diabetes, cancer, hypertension, heart, lung, and kidney diseases [4-6].

COVID-19 has rapidly become a global public health threat, endangering the health and well-being of all people and increasing social, political, religious, and socio-economic crises [7]. Numerous increases in COVID-19 cases and deaths continue to be reported in both developed and developing countries [8]. According to a World Health Organization (WHO) report on 20 September 2020, 30.6 million positive cases with 950,000 deaths worldwide have been recorded. On that date, Africa accounted for 1,145,397 COVID-19 positives with a total number of 24,757 deaths; and in Ethiopia, the number of COVID-19 cases reached 68,131 and confirmed deaths reached 1,089 [9].

SARS-CoV-2 appears to be transmitted from person to person through direct contact with the respiratory droplets, saliva, discharge from the nose when the infected person coughs, or sneezes, or indirectly through contaminated objects and surfaces as previously seen in SARS-CoV and MERS-CoV, the two other zoonotic coronaviruses [10, 11]. Implementation of stringent infection prevention and control (IPC) measures is the most effective way to reduce the spread of the COVID-19 [11]. WHO has strongly recommended that community members, especially those working in crowded areas such as food and drink establishments, to cover their nose and mouth with a tissue or elbow when coughing and sneezing, wear a mask, keep social distancing, perform appropriate alcohol-based hand rub (ARDS) and use appropriate personal protective equipment (PPE), all of which are simple and low-cost procedures against COVID-19 transmission as compared to its serious impact [12, 13].

Many factors can affect the practice of coronavirus infection prevention and control strategies such as availability of supplies, knowledge, attitude, training, and socio-demographic characteristics. Hence, to overcome the problem of COVID-19, all food workers should follow physical distancing guidance of at least 2 meters (6 feet), wear a face mask, and clean all surfaces with which employees and customers come into contact [14].

Failure to follow proper infection prevention and control strategies puts communities at risk. According to existing reports, despite the increment of COVID-19 prevalence in many developed and developing countries, the practice of IPC strategies among communities is not well studied[15]. Eastern Amhara, including the urban of Dessie City and Kombolcha Town, is one of the hotspot areas for COVID-19 due to the nearby road from Djibouti to Afar to Batti City to Kombolcha Town and then to Dessie City. Residents of both towns and travellers in Dessie City and Kombolcha Town use food and drink establishments, increasing the transmission of COVID-19. Implementation of IPC practices based on WHO and Ethiopian Ministry of Health (EMoH) guidelines is rapid, decisive, and collective action which saves millions of lives [13, 16, 17]. Therefore, this study was designed to assess the practice of infection prevention and control strategies against the novel coronavirus pandemic among food handlers in Dessie City and Kombolcha Town food and drinking establishments.

# **Methods and Materials**

## **Study Area**

The study was carried out in Dessie City and Kombolcha Town in South Wollo Zone. South Wollo Zone is one of the 13 zones within the Amhara regional state, Ethiopia. Dessie City and Kombolcha Towns are towns located 401km and 377 km away from Addis Ababa with an average elevation of 2510 and 1857 meters above sea level, respectively. The 2007 national census conducted by the [Central Statistical Agency](https://en.wikipedia.org/wiki/Central_Statistical_Agency_(Ethiopia)) [18] of Ethiopia reported that Dessie City had a total population of 151,174, of which 72,932 were male and 78,242 were female, whereas Kombolcha Town had 126,144 total populations of which 60,226 were male and 65,918 were female.

## **Study design, period, and population**

A facility-based cross-sectional study was conducted in July and August 2020 among food handlers in food and drinking establishments in Dessie City and Kombolcha Town. The source population was all food handlers working in Dessie City and Kombolcha Town food and drinking establishments. The study population was food handlers among the selected food and drinking establishments. Food and drinking establishments that had no food handlers during the data collection period were excluded.

## **Sample size determination and sampling techniques**

The sample size was determined using single population proportion formula with the assumptions of expected good infection prevention and control practice to prevent COVID-19 among food handlers 50% due to the absence of previous studies in a similar setting, Z_α/2_ value 1.96 at 95% confidence interval (CI) and 5% margin of error. After considering a 10% non-response rate from the calculated sample size of 384, the final sample size was 422. The total sample size was proportionally allocated for Dessie City and Kombolcha Town food and drinking establishments based on the total number of food and drinking establishments for each town. There were 349 and 204 food and drinking establishments (hotel, restaurant, bar and restaurant, cafeteria, and butcher houses) in Dessie City and Kombolcha Town, respectively. Therefore, the numbers of food and drinking establishments from the two towns by proportional allocation were 266 food and drinking establishments from Dessie City and 156 from Kombolcha Town.

The sampling units were food and drinking establishments that had at least one food handlers whose age 18 years and above. The sampling frame and source population was prepared by taking a list of food and drinking establishments that have at least food handlers (18 years and above) to be included in the sampling frame. Then, those eligible food and drinking establishments were coded to easily differentiate them during house-to-house visits at the time of data collection. Then, based on the source population (food handlers) for each *kebele,* the sample size was proportionally allocated for all ten *kebeles* based on their number of size of the food and drinking establishments. Using the sampling frame, a systematic random sampling with a fixed interval of every house was used to select a sampling unit within each *kebele.* To select the house paired with one eligible study participant, the data collector started with a benchmark of the known location and then walked straight forward to identify each house.

Furthermore, when more than one eligible study participant was present in the selected food and drinking establishments, a lottery method was used to select one study participant to estimate the proportion of good or poor practice of infection prevention and control in the study area relative to the total sample size. When one eligible study participant was not available from the selected food and drinking establishments, another visit was made the same day. If they were again not available, another visit was performed the next day to minimize the non-response rate. If not available after the three visits, it was taken as non-respondent.

## **Measurement of the outcome variable**

The outcome variable of this study was the good or poor practice of COVID-19 infection prevention and control among food handlers. Good infection prevention and control practices were determined for study participants who responded above or equal to the mean value among fifteen infection prevention and control practices questions, whereas poor infection prevention and control practices refer to those study participants who responded to fifteen infection prevention and control practices questions below the mean value.

## **Operational definitions**

**Infection prevention and control** is a scientific approach and practical solution designed to place barriers between a susceptible host and the microorganism [19].

**COVID-19** is a disease caused by a new SARS-CoV-2, a large family of transmissible viruses [20].

**Food handlers** are any person who directly handles packaged or unpackaged food, food equipment, and utensils, or food contact surfaces and is therefore expected to comply with food hygiene requirements [21].

**Food and drinking establishments** are an institution that provides food and drink services to a relatively large number of users [21].

**Good knowledge** refers to those study participants who correctly answered more than or equal to the mean score of eleven knowledge questions.

**Poor knowledge** refers to those study participants who answered less than the mean score of eleven knowledge questions

**Favourable attitude** refers to those study participants who scored greater than or equal to the mean score of nine attitude questions.

# **Unfavourable attitude** refers to those study participants who scored less than the mean score of nine attitude questions.

## **Data collection and quality control**

Data were collected using a structured pre-tested questionnaire and an on-the-spot observational checklist. The questionnaire and observational checklist were adapted from various reviewed literature, such as WHO and EMOH guidelines about COVID-19 infection prevention and control [7, 13, 17, 22]. The prepared English questionnaire was translated to Amharic (a local language) and then retranslated back to English by bilingual experts to ensure its consistency.

Four data collectors and two supervisors who were BSc. nurses and Environmental Health were recruited, respectively. All data collectors and supervisors had previous experience of COVID-19 data collection in a similar setting. The training was given to data collectors and supervisors by the principal investigator for two days about data collection procedures, contents of structured questionnaire and observational checklist with how to record data, how to keep social distance and wear a mask during an interview, and the ethical aspects of approaching the participants as well as the aim of the study. Data collectors kept at a social distance of at least 2 meters and wore a mask, and approached the participants politely and respectfully at the time of the interview. The supervisors monitored the data collection process daily; if a problem arose data collectors tried to solve it or they contacted the principal investigator by mobile or in person. The principal investigator and supervisors made daily spot checks for completeness of the questionnaire.

To assure the data quality, the questionnaire and observational checklist were pre-tested in 10 % of the selected food handlers at Haike town food and drinking establishments, an area not included in this study. Based on the pre-test result, amendments were done for unclear and vague questions. Each questionnaire and observational checklist was checked daily for completeness and any incomplete questionnaires or checklists were corrected in the same day by visiting the food and drinking establishments.

## **Data management and analysis**

The collected data were coded and entered into EpiData version 4.6 and exported to Statistical Package for Social Sciences (SPSS) version 25.0 for data cleaning and analysis. Mean with standard deviation were reported for continuous variables and for categorical variables, frequency with proportion was computed. Data were analyzed using a binary logistic regression model at 95% confidence interval and variables with *p*-value < 0.25 during the bivariate analysis were entered into a multivariable logistic regression analysis to see the relative effect of confounding variables and interaction of variables. Adjusted Odd ratio (AOR) with 95% CI was performed to determine the strength of association; variables with a *p*-value less than 0.05 were declared as statistically significant. **Hosmer-Lemeshow test was used to test** model fitness. Multicollinearity was tested using the Variance Inflation Factor (VIF) and tolerance test.

## **Ethical consideration**

Ethical clearance was obtained from the ethical review committee of Wollo University, College of Medicine and Health Sciences. A cooperation letter was given to the health bureau of Dessie City and Kombolcha Town and permission was obtained to start data collection. Written consent from owners /managers of the establishments and verbal consent from study subjects was obtained. Data collectors wore facemasks to prevent COVID-19 transmission to or from food handlers. Furthermore, social distancing was kept between data collectors and food handlers based on WHO recommended guidelines. Participants were informed of the objective of the study and assured that confidentiality would be kept. Data collection was conducted anonymously.

# **Results**

## **Socio-demographic characteristics of participants**

Of 422 planned participants, a total of 401eligible food handlers participated, for a response rate of 95%. In this study, more than half 217 (54.1%) of the participants were female. More than one-third 168 (41.9%) of food handlers were married, whereas about half 205 (51.1%) were single. The age of study participants ranged from 18 to 65 years with a median age of 26 years (IQR [interquartile range] = 23-32); most of the respondents were under 30 years 273 (68.1%). About half 207 (51.7%) of food handlers were waiters in the food and drinking establishments. Nearly half 198 (49.4%) of food and drinking establishments had 5-10 food handlers and 181 (45.2%) food handlers had an income of 27-54 $US (Table 1).

## **Availability of supplies**

Our results showed that about one-fifth 96 (23.9%) of the food and drinking establishments had written COVID-19 prevention guidelines, one-tenth 43(10.7%) of food and drinking establishments had a COVID-19 infection prevention and control focal person, more than one-third 166(41.4%) of food and drinking establishments had a specific budget for PPE and more than half 222 (55.4%) of food and drinking establishments had personal protective equipment against COVID-19 for their workers. The majority 362 (90.3%) of food and drinking establishments arranged infection prevention and control training for food handlers for the prevention of COVID-19 (Table2).

## **Knowledge about COVID-19 infection prevention and control strategies**

To determine the level of food handlers’ knowledge about COVID-19 infection prevention and control strategies, 11 questions that had yes/no alternatives were asked in the interviews. Our result showed that three-fourths (78.1%) (95% CI: 73.8 %-81.8%) of food handlers had good knowledge about infection prevention and control (Figure 1). The majority of food handlers 371 (92.5%) knew that all people are at risk of COVID-19 including themselves. About 358 (89.3%) food handlers knew that touching the nose, mouth, and eye is not recommended even if they used gloves when they gave service. Almost all 378 (94.3%) of food handlers knew that washing hands with soap, sanitiser and water would inhibit or kill the coronavirus. Most 375 (92.8%) of food handlers knew the importance of social distance and 371 (92.5%) of food handlers had been wearing PPE.

**Figure 1: Knowledge of COVID-19 infection prevention and control practices among food handlers in Dessie City and Kombolcha Town food and drinking establishments, Northeastern Ethiopia, July and August 2020.**

## **Attitude towards COVID-19 infection prevention and control practices**

Our finding showed that more than half 234 (58.4%) (95% CI: 53.6% - 63.6%) of food handlers had a favourable attitude about infection prevention and control practices (Figure 2). Most 318 (79.3%) study participants agreed that washing hands with soap or an alcohol-based antiseptic decreases the risk of transmission of COVID-19 while only 17 (4.2%) respondents strongly disagreed. Regarding social distancing, three-fourths 303 (75.6%) of food handlers agreed on its importance to reduce the transmission of novel coronavirus.

**Figure 2: Attitude about COVID-19 infection prevention and control practices among food handlers in Dessie City and Kombolcha Town food and drinking establishments, Northeastern Ethiopia, July and August 2020.**

## **Food handlers’ infection prevention and control strategy practice for COVID-19**

The mean (± standard deviation) score of practice questions was 13.12 ± 4.12. We found that more than one-third 176 (43.9%) (95% CI: 38.9-48.9) of food handlers had good COVID-19 infection prevention and control practice (Figure 3). Among all respondents, 365 (91.0%) reported that they washed their hands regularly, of whom 131 (35.9%) showed the practical hand washing techniques perfectly. Among the remaining 36 respondents who did not wash their hands regularly, the unavailability of handwashing materials was the important reason for not washing their hands. Most respondents 389 (97%) reported that when they sneezed, they covered their nose and mouth with an elbow. A majority 365 (91.0%) of food handlers had worn PPE, while the remaining 36 (9.0%) respondents did not wear PPE. Of 36 food handlers who did not wear PPE, 10 of them thought that wearing PPE is not always necessary to prevent COVID-19. Masks were worn by 351 (87.8%) food handlers.

**Figure 3: COVID-19 Infection prevention and control practices among food handlers in Dessie City and Kombolcha Town food and drinking establishments, Northeastern Ethiopia, July and August 2020.**

## **Bivariate and multivariable analysis**

From the bivariate analysis age, educational status, years of experience, availability of COVID-19 prevention guidelines, availability of COVID-19 infection prevention and control focal person, availability of specific budget for COVID-19 infection prevention and control, availability of PPE in food and drinking establishments, ever having taken COVID-19 infection prevention and control training and attitude on COVID-19 infection prevention and control were *p*-value <0.25 and retained into the multivariable analysis.

From the multivariable analysis, respondents who had an educational status of college or above were almost twice as likely to have good practise than those who were illiterate (AOR=1.97;95% CI:1.32–3.75). The odds of having good COVID-19 infection prevention and control practice among food handlers who had work experience of greater than 5 years were 3 times (AOR=2.55; CI:1.43-5.77) greater compared to those who had 5 or fewer years of work experience. Those food handlers who had written COVID-19 prevention guidelines in their working establishment were almost 3 times more likely to practice COVID-19 infection prevention and control strategies than those who did not (AOR=2.68; 95% CI: 1.52-4.75). Food handlers who had taken COVID-19 infection prevention and control training were 3 times more likely to have good COVID-19 infection prevention and control practice than those who had not taken it (AOR=3.26;95% CI: 1.61-6.61) (Table 3).

# **Discussion**

We conducted a cross-sectional study to assess the level of infection prevention and control practices to prevent COVID-19 among food handlers in Dessie City and Kombolcha town food and drinking establishments, in Northeastern Ethiopia. Out of 401 food handlers, 78.1% had good knowledge about COVID-19 infection prevention and control strategy practices. Regarding the attitude of food handlers about COVID-19 infection prevention and control strategy practices, 58.4% had a favourable attitude. We found that educational status, years of experience, availability of COVID-19 infection prevention and control guidelines and ever having taken COVID-19 infection prevention and control training had a statistically significant association with the food handlers’ practice of COVID-19 infection prevention and control strategies.

In this study, 43.9% of the food handlers had good COVID-19 infection prevention and control practices. Our study finding was consistent with previous studies in Addis Zemen, Ethiopia (47.3%) [6] and Bangladesh (44.8%) [23]. This finding was lower than reported by studies in Amhara region (62%) [24], Uganda (74%) [25], China (89.7%) [26] and Pakistan (88.7%) [27]. The possible reasons for this discrepancy might include the difference in the socioeconomic status, culture, and educational level of study participants. On the other hand, the current study finding was higher than reported by studies conducted in Bale Zone, Ethiopia (36.8%) [28]  and the Philippines (32.4%) [29]. This may be due to the differences in the tool used for assessment of COVID-19 infection prevention and control practice, differences in study participants where our study focused on food handlers, or a methodological difference such as the difference in the accessibility of COVID-19 infection prevention and control guidelines and types of PPE in the food and drinking establishments.

# Though the execution was questionable, most of the food handlers tried to follow Ethiopia's Ministry of Health and WHO infection prevention and control practice recommendations. These include regular wearing of PPE including a face mask, hand hygiene, and social distancing. About 91% of food handlers reported wearing PPE, of whom 87.8% had worn a face mask. Ninety-one percent and 83.8% of participants reported washing hands regularly and keeping social distance one meter apart, respectively. These findings were supported by a study at Jimma University Medical Center in Ethiopia [30]. In contrast, a study conducted in Mizan-Aman Ethiopia revealed that about 81.8% of waiters did not wash their hands frequently[31].

The overall prevention knowledge towards the COVID-19 pandemic was in line with an online survey conducted in Ethiopia [32], studies conducted in Jordan [33] and Nigeria [34]. This finding indicated the significance of enhancing COVID-19 knowledge through different techniques as a factor to improve the preventive strategies against the disease. Food handlers need to be encouraged to improve their attitude towards infection prevention and control against COVID 19 virus. The current study finding of attitude is similar to that found in studies conducted in northern Ethiopia [35] and China [36].

This study identified factors significantly associated with COVID-19 infection prevention and control practice. Higher educational status was significantly associated with good practice of infection prevention and control among food handlers. This finding was supported by studies conducted in Addis Zemen, Ethiopia [6], Mizan-Aman Ethiopia [31], Uganda [25], Iran [37], and China [38]. The probable reasons for this finding are that educated food handlers may have a good understanding, perception, knowledge, and skill about COVID-19 infection prevention and control measures; also, they may easily access the recommendations of health care professionals about COVID-19 preventive measures. Having long work experience was associated with good COVID-19 infection prevention and control practice among food handlers. This finding was supported by studies in Pakistan [27] and China [26]. The possible explanation is that a difference in the understanding of the importance of COVID-19 infection prevention and control between inexperienced and more senior food handlers. As food handlers work experience increases and they face challenges due to impacts of poor infection prevention and control practice, their attitude about the benefit of infection prevention and control implementation improves over time. It might also be that as workers food handling skills increase, it is easier for them to remember and perform the extra tasks required for infection prevention and control even when they are busy.

Availability of written COVID-19 guidelines increased good COVID-19 infection prevention and control practices. This finding was similar to that of studies in the Amhara region [24] and South Africa [39]. This could be because standardized COVID-19 prevention guidelines are used as the source of information about how to practice COVID-19 infection prevention and control strategies that reduce transmission. Also, food handlers may become alarmed when they see COVID-19 prevention guidelines in the catering area and be motivated to become familiar with the guidelines.

The main purpose of COVID-19 infection prevention and control training was to demonstrate food and drink establishments' commitment to the well-being of customers and staff. In this study, food handlers who had taken COVID-19 infection prevention and control training were more likely to have good COVID-19 infection prevention and control practice than those who had not taken it. This result was supported by studies conducted in the Amhara region [24], Uganda [25], and China [40]. This might be because training can impact knowledge about the COVID-19, equip and update food handlers about COVID-19 infection prevention precautions and enhance skills in how to use PPE and implement infection prevention and control guidelines.

A weakness of our study may be that of social desirability bias during self-reporting; however, we tried to control social desirability bias by the employment of proxy data. Furthermore, the level of accuracy of the measuring instrument was revised after pre-testing the questionnaire data collection tools. The findings of this study may not be represented at the national level, as the study was conducted only in Dessie City and Kombolcha Towns food and drinking establishments.

# **Conclusion**

This study revealed that more than one-third of food handlers had good infection prevention and control practices. Our result also showed that three-fourths and more than half of food handlers had good knowledge and favourable attitude about infection prevention and control, respectively. Factors significantly associated with good infection prevention and control practices included higher educational status, more years of experience, availability of WASH- infection prevention and control guidelines and training. We recommend that integrated work is urgently needed to improve good practice, knowledge, and attitude about infection prevention and control among food handlers through providing them with health education and training aimed at controlling the transmission of COVID-19 between food handlers and customers. We also recommend that more studies be conducted on a larger scale, especially at regional and national levels.

# **Acknowledgements**

We would like to thank Dessie City Health Bureau for the support we received during data collection. Our gratitude also goes to Dessie City and Kombolcha Town culture and tourism offices for providing the necessary data during the proposal development and data collection period. We also highly appreciate the owners of Dessie City and Kombolcha Town food and drinking establishments for their support and the information they provided when we needed it. We also extend our gratitude to food handlers, data collectors, and supervisors for their valuable time and cooperation during the study. Last but not the least, we also thank Lisa Penttila for the language editing of the manuscript.

# **Abbreviations**

AOR: adjusted odds ratio; CI: confidence interval; COVID: coronavirus infectious disease; PPE: personal protective equipment; WHO: World Health Organization

# **References**

1.	Huang C, Wang Y, Li X, Ren L, Zhao J, Hu Y, Zhang L, Fan G, Xu J, Gu X: Clinical features of patients infected with 2019 novel coronavirus in Wuhan, China. The lancet 2020, 395(10223):497-506.

2.	Organization WH: Coronavirus disease 2019 (COVID-19): situation report, 70. 2020.

3.	Organization WH: Infection prevention and control guidance for long-term care facilities in the context of COVID-19: interim guidance, 21 March 2020. In.: World Health Organization; 2020.

4.	Wu J, Mafham M, Mamas M, Rashid M, Kontopantelis E, Deanfield J, de Belder M, Gale CP: Place and underlying cause of death during the COVID19 pandemic: a retrospective cohort study of 3.5 million deaths in England and Wales, 2014 to 2020. medRxiv 2020.

5.	Wu C, Chen X, Cai Y, Zhou X, Xu S, Huang H, Zhang L, Zhou X, Du C, Zhang Y: Risk factors associated with acute respiratory distress syndrome and death in patients with coronavirus disease 2019 pneumonia in Wuhan, China. JAMA internal medicine 2020.

6.	Akalu Y, Ayelign B, Molla MD: Knowledge, attitude and practice towards COVID-19 among chronic disease patients at Addis Zemen Hospital, Northwest Ethiopia. Infection and drug resistance 2020, 13:1949.

7.	Walker P, Whittaker C, Watson O, Baguelin M, Ainslie K, Bhatia S, Bhatt S, Boonyasiri A, Boyd O, Cattarino L: Report 12: The global impact of COVID-19 and strategies for mitigation and suppression. 2020.

8.	Tsai J, Wilson M: COVID-19: a potential public health problem for homeless populations. The Lancet Public Health 2020, 5(4):e186-e187.

9.	Organization WH: Coronavirus disease (‎ COVID-19)‎. 2020.

10.	NUMBERS SI, ASSESSMENT WR: Coronavirus disease 2019 (COVID-19). Americas 2020, 10(2):1.

11.	Rothan HA, Byrareddy SN: The epidemiology and pathogenesis of coronavirus disease (COVID-19) outbreak. Journal of autoimmunity 2020:102433.

12.	Organization WH: Rational use of personal protective equipment for coronavirus disease (COVID-19): interim guidance, 27 February 2020. In.: World Health Organization; 2020.

13.	Organization WH: Infection prevention and control during health care when novel coronavirus (‎‎‎‎‎‎ nCoV)‎‎‎‎‎‎ infection is suspected: interim guidance, 25 January 2020. 2020.

14.	COVID W: and food safety: Guidance for food businesses. 2020. In.

15.	Allegranzi B, Nejad SB, Combescure C, Graafmans W, Attar H, Donaldson L, Pittet D: Burden of endemic health-care-associated infection in developing countries: systematic review and meta-analysis. The Lancet 2011, 377(9761):228-241.

16.	Organization WH: Rational use of personal protective equipment (PPE) for coronavirus disease (COVID-19): interim guidance, 19 March 2020. In.: World Health Organization; 2020.

17.	Baye K: COVID-19 prevention measures in Ethiopia: Current realities and prospects, vol. 141: Intl Food Policy Res Inst; 2020.

18.	Csace I: Ethiopia demographic and health survey 2016. Addis Ababa, Ethiopia, and Rockville, Maryland, USA: CSA and ICF 2016.

19.	Organization WH: Improving infection prevention and control at the health facility: interim practical manual supporting the implementation of the WHO Guidelines on Core Components of Infection Prevention and Control Programmes. In.: World Health Organization; 2018.

20.	Sohrabi C, Alsafi Z, O’Neill N, Khan M, Kerwan A, Al-Jabir A, Iosifidis C, Agha R: World Health Organization declares global emergency: A review of the 2019 novel coronavirus (COVID-19). International Journal of Surgery 2020.

21.	Meleko A, Henok A, Tefera W, Lamaro T: Assessment of the sanitary conditions of catering establishments and food safety knowledge and practices of food handlers in Addis Ababa University Students’ Cafeteria. Science 2015, 3(5):733-743.

22.	Sahiledengle B, Gebresilassie A, Getahun T, Hiko D: Infection prevention practices and associated factors among healthcare workers in governmental healthcare facilities in Addis Ababa. Ethiopian Journal of health sciences 2018, 28(2):177-186.

23.	Paul A, Sikdar D, Hossain MM, Amin MR, Deeba F, Mahanta J, Jabed MA, Islam MM, Noon SJ, Nath TK: Knowledge, attitudes, and practices toward the novel coronavirus among Bangladeshis: Implications for mitigation measures. PloS one 2020, 15(9):e0238492.

24.	Asemahagn MA: Factors determining the knowledge and prevention practice of healthcare workers towards COVID-19 in Amhara region, Ethiopia: a cross-sectional survey. Tropical medicine and health 2020, 48(1):1-11.

25.	Olum R, Chekwech G, Wekha G, Nassozi DR, Bongomin F: Coronavirus Disease-2019: Knowledge, Attitude, and Practices of Health Care Workers at Makerere University Teaching Hospitals, Uganda. Frontiers in Public Health 2020, 8:181.

26.	Zhou M, Tang F, Wang Y, Nie H, Zhang L, You G, Zhang M: Knowledge, attitude and practice regarding COVID-19 among health care workers in Henan, China. Journal of Hospital Infection 2020.

27.	Saqlain M, Munir M, Rehman S, Gulzar A, Naz S, Ahmed Z, Tahir A, Mashhood M: Knowledge, attitude, practice and perceived barriers among healthcare workers regarding COVID-19: a cross-sectional survey from Pakistan. Journal of Hospital Infection 2020, 105(3):419-423.

28.	Zenbaba D, Sahiledengle B, Bogale D: Practices of Healthcare Workers regarding Infection Prevention in Bale Zone Hospitals, Southeast Ethiopia. Advances in Public Health 2020, 2020.

29.	Lau LL, Hung N, Go, D. J, Ferma J, Choi M, Dodd W, Wei X: Knowledge, attitudes and practices of COVID-19 among income-poor households in the Philippines: A cross-sectional study. Journal of global health 2020, 10(1).

30.	Kebede Y, Yitayih Y, Birhanu Z, Mekonen S, Ambelu A: Knowledge, perceptions and preventive practices towards COVID-19 early in the outbreak among Jimma university medical centre visitors, Southwest Ethiopia. PloS one 2020, 15(5):e0233744.

31.	Qanche Q, Asefa A, Nigussie T, Hailemariam S, Duguma T: Knowledge of COVID-19 and preventive behaviors among waiters working in food and drinking establishments in Southwest Ethiopia. Plos one 2021, 16(1):e0245753.

32.	Bekele D, Tolossa T, Tsegaye R, Teshome W: The knowledge and practice towards COVID-19 pandemic prevention among residents of Ethiopia. An online cross-sectional study. Plos one 2021, 16(1):e0234585.

33.	Olaimat AN, Aolymat I, Shahbaz HM, Holley RA: Knowledge and information sources about COVID-19 among university students in Jordan: a cross-sectional study. Frontiers in public health 2020, 8:254.

34.	Reuben RC, Danladi MM, Saleh DA, Ejembi PE: Knowledge, attitudes and practices towards COVID-19: an epidemiological survey in North-Central Nigeria. Journal of community health 2020:1-14.

35.	Tadesse DB, Gebrewahd GT, Demoz GT: Knowledge, attitude, practice and psychological response toward COVID-19 among nurses during the COVID-19 outbreak in northern Ethiopia, 2020. New Microbes and New Infections 2020, 38:100787.

36.	Ma L, Liu H, Tao Z, Jiang N, Wang S, Jiang X: Knowledge, Beliefs/Attitudes, and practices of rural residents in the prevention and control of COVID-19: an online questionnaire survey. The American journal of tropical medicine and hygiene 2020, 103(6):2357-2367.

37.	Honarvar B, Lankarani KB, Kharmandar A, Shaygani F, Zahedroozgar M, Haghighi MRR, Ghahramani S, Honarvar H, Daryabadi MM, Salavati Z: Knowledge, attitudes, risk perceptions, and practices of adults toward COVID-19: a population and field-based study from Iran. International journal of public health 2020, 65(6):731-739.

38.	Zhong B-L, Luo W, Li H-M, Zhang Q-Q, Liu X-G, Li W-T, Li Y: Knowledge, attitudes, and practices towards COVID-19 among Chinese residents during the rapid rise period of the COVID-19 outbreak: a quick online cross-sectional survey. International journal of biological sciences 2020, 16(10):1745.

39.	Cooper S, Wiyeh A, Schmidt B-M, Wiysonge CS: Cochrane corner: factors that influence compliance by healthcare workers with infection prevention and control guidelines for COVID-19 and other respiratory infections. The Pan African Medical Journal 2020, 35(23).

40.	Yu J, Ding N, Chen H, Liu X-J, He W-j, Dai W-c, Zhou Z-g, Lin F, Pu Z-h, Li D-f: Infection control against COVID-19 in departments of radiology. Academic Radiology 2020.

**Table 1: Bivariate analysis of socio-demographic characteristics of food handlers associated with infection prevention and control practice in Dessie City and Kombolcha Town food and drinking establishments, Northeastern Ethiopia, July – August 2020**

| **Variables** | **Category** | **Frequency (*N* = 401)** | **Infection prevention and control practice status** | | **COR (95% CI)** | ***p*-value** |
| --- | --- | --- | --- | --- | --- | --- |
|  |  |  | **Good** | **Poor** |  |  |
|  |  | **n (%)** | **n** | **n** |  |  |
| Age of food handlers (in years) | 18-30 | 273(68.1) | 95 | 178 | Ref |  |
|  | 31-49 | 114(28.4) | 71 | 43 | 3.09(1.97-4.87) | < 0.001 |
|  | >=50 | 14(3.5) | 10 | 4 | 4.68(1.43-15.33) | 0.011 |
| Sex | Male | 184(45.9) | 85 | 99 | 1.19(0.80-176) | 0.392 |
|  | Female | 217(54.1) | 91 | 126 | Ref |  |
| Marital status | Married | 168(41.9) | 89 | 79 | Ref |  |
|  | Single | 205(51.1) | 75 | 130 | 0.51(0.34-0.78) | 0.252 |
|  | Divorced | 17(4.2) | 6 | 11 | 0.48(0.17-1.37) | 0.372 |
|  | Widowed | 11(2.8) | 6 | 5 | 1.07(0.31-3.63) | 0.920 |
| Educational status | Illiterate | 19(4.7) | 12 | 7 | Ref |  |
|  | Informal Education | 19(4.7) | 9 | 10 | 1.54(0.75-2.66) | 0.330 |
|  | Primary | 65(16.2) | 27 | 38 | 1.80(0.93-2.71) | 0.102 |
|  | Secondary | 207(51.7) | 68 | 139 | 2.64(1.42-5.03) | 0.012 |
|  | College or above | 91(22.7) | 60 | 31 | 2.07(1.52–3.95) | 0.003 |
| Job position in the food and drinking establishments | Cook | 101(25.2) | 51 | 50 | Ref |  |
|  | Dish washer | 62(15.5) | 30 | 32 | 2.39(1.23-4.65) | 0.767 |
|  | Waiter | 207(51.7) | 43 | 164 | 2.12(1.12-4.03) | 0.310 |
|  | Others | 31(7.8) | 18 | 13 | 2.79(1.74-4.47) | 0.650 |
| Number of food handlers in each food and drinking establishment (persons) | <5 | 85(21.2) | 35 | 50 | Ref |  |
|  | 5-10 | 198(49.4) | 69 | 129 | 0.76(0.45-1.29) | 0.312 |
|  | 11-15 | 63(15.7) | 32 | 31 | 1.48(0.77-2.84) | 0.246 |
|  | >15 | 55(13.7) | 40 | 15 | 3.81(1.83-7.94) | 0.302 |
| Years of service | <1 | 157(39.2) | 53 | 104 | 0.66(0.43-1.03) | 0.267 |
|  | 1-5 | 196(48.8) | 85 | 111 | Ref |  |
|  | > 5 | 48(12) | 38 | 10 | 4.96(2.34-10.52) | < 0.001 |
| Food handlers’ monthly salary (USD [United States Dollars], $)* | <28 | 86(21.4) | 13 | 73 | Ref |  |
|  | 28-56 | 181(45.2) | 79 | 102 | 4.35(2.25-8.41) | 0.251 |
|  | > 56 | 134(33.4 | 84 | 50 | 9.43(4.75-18.73) | 0.410 |

## *Average exchange rate of 1 $US to Ethiopian Birr (ETB) in July and August 2020 was 1 $US = 35.6395 ETB

**Table 2: Bivariate analysis of availability of supplies associated with infection prevention and control practice in Dessie City and Kombolcha Town food and drinking establishments, Northeastern Ethiopia, July – August 2020**

| **Variables** | **Response** | **Frequency (*N*= 401)** | **Infection prevention and control practice status** | | **COR (95% CI)** | ***p*-value** |
| --- | --- | --- | --- | --- | --- | --- |
|  |  |  | **Good** | **Poor** |  |  |
|  |  | **n (%)** | **N** | **n** |  |  |
| Availability of COVID 19 prevention guidelines in food and drinking establishments | No | 305(76.1) | 110 | 195 | Ref |  |
|  | Yes | 96(23.9) | 66 | 30 | 3.90(2.39-6.37) | <0.001 |
| Availability of COVID 19 infection prevention and control focal person | No | 358(89.3) | 142 | 216 | Ref |  |
|  | Yes | 43(10.7) | 34 | 9 | 5.75(2.68-12.35) | 0.010 |
| Availability of specific budget for PPE in this COVID-19 era | No | 235(58.6) | 74 | 161 | Ref |  |
|  | Yes | 166(41.4) | 102 | 64 | 3.47(2.29-5.26) | 0.011 |
| Availability of personal protective equipment | No | 179(44.6) | 65 | 114 | Ref |  |
|  | Yes | 222(55.4) | 111 | 111 | 1.75(1.17-2.62) | 0.006 |
| COVID-19 infection prevention training arranged for food handlers | No | 39(9.7) | 13 | 26 | Ref |  |
|  | Yes | 362(90.3) | 163 | 199 | 1.64(0.82-3.29) | 0.265 |
| Posted information related to COVID-19 including emergency phone numbers on visible sites | No | 275(68.6) | 87 | 188 | Ref |  |
|  | Yes | 126(31.4) | 89 | 37 | 3.20(3.28-8.23) | 0.265 |
| Services provided in accordance with COVID-19 safety measures | No | 80(20.0) | 29 | 51 | Ref |  |
|  | Yes | 321(80.0) | 147 | 174 | 1.49(0.90-2.46) | 0.456 |
| Availability of registration book for documenting events related to COVID-19 | No | 346(86.3) | 139 | 207 | Ref |  |
|  | Yes | 55(13.7) | 37 | 18 | 3.06(1.68-5.60) | 0.650 |
| Order servants and customers to practice social distancing and to avoid touch each other | No | 91(22.7) | 25 | 66 | Ref |  |
|  | Yes | 310(77.3) | 151 | 159 | 2.51(1.50-4.18) | 0.901 |
| Frequently washing and immersing table cloths in a mixture of one part bleach and nine parts water for 10 minutes and finally rinsing with pure water. | No | 286(71.3) | 108 | 178 | Ref |  |
|  | Yes | 115(28.7) | 68 | 47 | 2.39(1.53-3.71) | 0.451 |
| Having well cleaned and ventilated service rooms, toilets, meeting halls, and corridors | No | 128(31.9) | 46 | 82 | Ref |  |
|  | Yes | 273(68.1) | 130 | 143 | 1.62(1.05-2.50) | 0.329 |
| Daily cleaning of doors, walls, windows, tables, chairs, and mobile phones by sanitizer or a solution containing one part bleach and nine parts water | No | 127(31.7) | 60 | 67 | Ref |  |
|  | Yes | 274(68.3) | 116 | 158 | 0.82(0.54-1.25) | 0.357 |
| Treating materials and equipment used by customers with a solution containing one part bleach and nine parts water | No | 273(68.1) | 96 | 177 | Ref |  |
|  | Yes | 128(31.9) | 80 | 48 | 3.10(1.99-4.75) | 0.452 |
| Rinsing brooms, brushes, and utility gloves with bleach | No | 152(37.9) | 59 | 93 | Ref |  |
|  | Yes | 249(62.1) | 117 | 132 | 1.40(0.93-2.11) | 0.311 |
| Chair arrangement is 2 meters apart | No | 193(48.1) | 61 | 132 | Ref |  |
|  | Yes | 208(51.9) | 115 | 93 | 2.68(1.78-4.03) | 0.271 |
| Accessibility of covered dust bins in each room | No | 81(20.2) | 30 | 51 | Ref |  |
|  | Yes | 320(79.8) | 146 | 174 | 1.43(0.86-2.36) | 0.264 |
| Availability of segregating materials to separate dry and liquid waste | No | 69(17.2) | 34 | 35 | Ref |  |
|  | Yes | 332(82.8) | 142 | 190 | 0.77(0.46-1.29) | 0.323 |
| Collecting and disposing of wastes properly | No | 63(15.7) | 29 | 34 | Ref |  |
|  | Yes | 338(84.3) | 147 | 191 | 0.90(0.53-1.55 | 0.709 |
| Ever taken COVID-19 infection prevention and control training | No | 343(85.5) | 132 | 211 | Ref |  |
|  | Yes | 58(14.5) | 44 | 14 | 5.02(2.65-9.52) | <0.001 |

**Table 3: Multivariable analysis of factors associated with COVID-19 infection prevention and control practice among food handlers in Dessie City and Kombolcha Town food and drinking establishments, Northeastern Ethiopia, July – August 2020**

| **Variables** | | **Infection prevention and control practice status** | | **AOR (95% CI)** | ***P*-value** |
| --- | --- | --- | --- | --- | --- |
|  |  | **Good (n)** | **Poor (n)** |  |  |
| Educational status | Illiterate | 7 | 12 | Ref |  |
|  | Informal education | 10 | 9 | 1.32(0.50-2.10) |  |
|  | Primary school | 36 | 29 | 1.56(0.86-2.37) |  |
|  | Secondary school | 141 | 66 | 2.40(1.22-4.73) |  |
|  | College or above | 60 | 31 | 1.97(1.32–3.75) | 0.042 |
| Years of experience | <1 | 53 | 104 | 0.84(0.51-1.38) | 0.678 |
|  | 1-5 | 85 | 111 | Ref |  |
|  | >5 | 38 | 10 | 2.55(1.43-5.77) | 0.025 |
| Availability of COVID-19 infection prevention and control guidelines in food and drinking establishments | Yes | 66 | 30 | 2.68(1.52-4.75) | < 0.001 |
|  | No | 110 | 195 | Ref |  |
| Ever taken infection prevention and control training to prevent COVID-19 | Yes | 44 | 14 | 3.26(1.61-6.61) | < 0.001 |
|  | No | 132 | 211 | Ref |  |

Ref, reference category; AOR, adjusted odds ratio
