# Supplementary material for: COVID-19 infection prevention practices among a sample of food handlers of food and drink establishments in Ethiopia
Source: PLoS One. 2022 Jan 24;17(1):e0259851. doi: 10.1371/journal.pone.0259851 (PMC8786123; doi:10.1371/journal.pone.0259851)
Supplement: Attachment — Submitted filename: Response to reviwers.docx [file pone.0259851.s007.docx]

**Rebuttal letter**

**Response to reviewers for the paper PONE-D-21-12395R1**

**Dear reviewers;**

**Thank you for your valuable comments and we updated the manuscript accordingly.**

**Here are the detail answers for your concerns:**

**Reviewer #2:**

The authors responded to my suggestion on considering a higher cut-off score to define "good", that the mean is typically the best measure of central tendency because it takes all values into
account (the distribution of the data is normal). I believe the authors may have misunderstood what I meant.

If the maximum possible score was 100, and the average mean score was just 20, it is not reasonable to assume that those who scored above 20 are all considered to be "good". However, if the mean score was 90 out of 100, potentially those who scored below 90 but say above 70 would also be considered to have done well. So the mean is not a good way to
define "good".

The definition of good should be based on a proportion of the maximum possible score. I think 70 out of 100 is considered "good". Possibly 60 out of 100 could also be "good". I acknowledge that there may be no studies out there which can be referenced to set the threshold definition for good. But surely, 50% cannot be considered "good".

I urge the authors to consider setting the threshold for the definition of "good" at 70% (which would be a score of 18.75) or at 60% (which would be a score of 15). Otherwise, the authors would have to amend their manuscript throughout to state that they "analyzed factors associated with participants who scored above the mean.", which in no way suggests whether these are considered "good" or otherwise.

**Response: We appreciate your valuable comment; we accept the comment and made a correction as suggested by taking 70% of knowledge questions as a cut-off point to say good and poor. Please see in find page 9, line number 162-165 of the manuscript.**

**Reviewer #4:**

**Question:** This manuscript has not been prepared from the right perspective of the research methodology. I don’t believe that the sample size calculation is relevant as the underlying primary aim is the multivariate regression modeling

**Response: Your concern is valid if we were used a multilevel analysis to identify individual, household and community level factors. However, our study is a simple cross-sectional study with a binary outcome of Good or Poor preventive practices. The multivariable analysis is used to control the confounders. But we did not use multivariate regression model, which is a different concept compared to multivariable analysis. Thus, we believe that determining the sample size using single proportion sample size determination method is valid in our case and the sample size 422 food handlers as a sample size for this cross-sectional study is enough.**

**Question:** Dichotomising the fifteen-infection scale at an arbitrary cut-off (suing the sample mean) reduces the statistical power by the same amount as would discarding a third of the data (1, 2). In general, there is no good reason to believe that there is an underlying dichotomy, and if one exists there is no reason why it should be at the mean especially as the fifteen-infection scale is not normally distributed. The fifteen-infection scale looks like an ideal candidate for one of the ordinal cumulative probability models. Therefore, I suggest using the proportional odds model.

**Response: We would like to thank the reviewer for this important comment. We accept your comment and re-dichotomized the infection prevention practices questions using 75% as a cutoff point. Good infection prevention practice was determined for food handlers who scored 75% and above, whereas poor infection prevention practices refers to those food handlers who scored below 75% of practice questions. Please refer page 8, line number 149-151 of the main document.**

**Reviewer #6: Dear authors,**

Thank you for allowing me to review this manuscript. I think this is a good study and worth for publication. Generally, I am satisfied with all the corrected version from the manuscript. Keep up the good work. Thanks

**Response: We duly appreciate your positive scientific remark for our effort. Thank you.**

**Reviewer #7:**

The article seems clearer with the changes you have made. Thank you for taking my suggestions into consideration.

**Response: Thank you.**

________________________________

**Thank you for your time in reviewing our paper and we hope that the revised version is acceptable for publication in PLoS ONE.**

**Sincerely yours,**

**Dr Metadel Adane (PhD in Water and Public Health)**
