# Supplementary material for: COVID-19 infection prevention practices among a sample of food handlers of food and drink establishments in Ethiopia
Source: PLoS One. 2022 Jan 24;17(1):e0259851. doi: 10.1371/journal.pone.0259851 (PMC8786123; doi:10.1371/journal.pone.0259851)
Supplement: Attachment — Submitted filename: Response to reviewers.docx [file pone.0259851.s008.docx]

**Rebuttal letter**

**Response to Editor questions/comments for the paper PONE-D-21-12395R2**

**Dear Editor,**

**Thank you for your pertinent comments and we updated the manuscript accordingly.**

**Here are the detail answers for your concerns:**

**Additional Editor Comments (if provided):**

Great effort was made by the authors to utilize the feedback that was provided for them to correct for resubmission after revision. Meanwhile, minor modifications are required according to PLOS one guidelines:

**Response: Thank you for your positive remark and for your decision of minor revision required. We updated the manuscript accordingly and please find the detail response for each hereunder.**

1. Please consider restricting the Abstract to no more than 300 words

**Response: Thank you. We updated the manuscript to 300 words.**

2. The authors declared that oral consent was sought from participants before the study started, please justify why authors did not take written consent and please clarify whether the institutional review board approved the use of verbal consent.

**Response: Sorry for this confusion. We used written informed consent, not oral consent. We updated the manuscript.**

3. Please also state in the ethics approval the approval number

**Response: Thank you for this important comment. We included the approval number in the ethics statement.**
